# Immune niches orchestrated by intestinal mesenchymal stromal cells lining the crypt-villus

**DOI:** 10.3389/fimmu.2022.1057932

**Published:** 2022-11-03

**Authors:** Hongxiang Sun, Jianmei Tan, Hongqian Chen, Ningbo Wu, Bing Su

**Affiliations:** ^1^ Shanghai Institute of Immunology, Department of Immunology and Microbiology, and the Ministry of Education Key Laboratory of Cell Death and Differentiation, Shanghai Jiao Tong University School of Medicine, Shanghai, China; ^2^ Department of Gastroenterology, Center for Immune-Related Diseases, Ruijin Hospital, Shanghai Jiao Tong University School of Medicine, Shanghai, China; ^3^ Shanghai Jiao Tong University School of Medicine–Yale Institute for Immune Metabolism, Shanghai Jiao Tong University School of Medicine, Shanghai, China; ^4^ Key Laboratory of Molecular Radiation Oncology of Hunan Province, Xiangya Hospital, Central South University, Changsha, China; ^5^ Academy of Integrative Medicine, Shanghai University of Traditional Chinese Medicine, Shanghai, China

**Keywords:** kintestinal mesenchymal stromal cell1, crypt-villus axis2, stromal-immune interaction3, MRISC4, telocytes5

## Abstract

The mammalian intestine is an organ that can be spatially defined by two axes: longitudinal and vertical. Such anatomical structure ensures the maintenance of a relatively immuno-quiescent and proliferation-promoting crypt for intestinal stem cell differentiation while actively warding off the invading intestinal microbes at the villus tip during digestion and nutrient absorption. Such behavior is achieved by the fine coordination among intestinal epithelial cells, intestinal mesenchymal stromal cells and tissue-resident immune cells like myeloid cells and lymphocytes. Among these cell types resided in the colon, intestinal mesenchymal stromal cells are considered to be the essential link between epithelium, vasculature, neuronal system, and hematopoietic compartment. Recent advancement of single cell and spatial transcriptomics has enabled us to characterize the spatial and functional heterogeneity of intestinal mesenchymal stromal cells. These studies reveal distinctive intestinal mesenchymal stromal cells localized in different regions of the intestine with diverse functions including but not limited to providing cytokines and growth factors essential for different immune cells and epithelial cells which predict niche formation for immune function from the villus tip to the crypt bottom. In this review, we aim to provide an overall view of the heterogeneity of intestinal mesenchymal stromal cells, the spatial distribution of these cells along with their interaction with immune cells and the potential regulatory cytokine profile of these cell types. Summarization of such information may enrich our current understanding of the immuno-regulatory functions of the newly identified mesenchymal stromal cell subsets beyond their epithelial regulatory function.

## 1 Introduction

Mesenchymal stromal cells, especially fibroblasts are a group of non-epithelial, non-hematopoietic, non-endothelial, non-neuronal cells that produce growth factors, cytokines, and extracellular matrices for the normal growth and development of multiple organs (1). Recent studies utilized single cell transcriptomics have revealed the cellular heterogeneity of fibroblasts across human and mouse (2). Those cells include *Pi16* ^+^ adventitial fibroblasts (3), *Col15a1* ^+^ parenchymal fibroblasts (4), *Ccl19* ^+^ fibroblastic reticular cells (5), and other organ specific fibroblast subsets like *Cxcl12* ^+^ mesenchymal stromal cells (6), *Npnt* ^+^ alveolar fibroblasts, *Comp* ^+^ arterial fibroblasts, *Wt1* ^+^ red pulp fibroblasts (7), *Hhip* ^+^ fibroblasts, *Pdgfra* ^lo^ intestinal fibroblasts and *Pdgfra* ^Hi^ intestinal telocytes (8). In human, on the other hand, there are also 5 major subsets of intestinal fibroblasts encompassing from *PI16* ^+^ adventitial fibroblasts, *NPNT* ^+^ alveolar fibroblasts, *ADAMDEC1* ^+^ intestinal fibroblasts, *CCL19* ^+^ lymphoid organ fibroblasts, and *LRRC15* ^+^ cancer associated fibroblasts (2, 9). Those mesenchymal stromal cells or fibroblasts can regulate tissue immunity *via* multiple mechanisms including cytokines, chemokines, adhesion molecules and immuno-regulatory metabolites in addition to facilitation of structural development *via* providing micro-environment for maintenance and differentiation of epithelial cells, endothelial cells or neuronal cells. Once the balanced interaction among those mesenchymal stromal cells has been interfered, the mesenchymal stromal cells would be activated and get involved in multiple different diseases including inflammatory diseases, cancer, fibrosis, cardiovascular diseases, congenital disorder, etc. Therefore, mesenchymal stromal cells, by strategically reside in a position that links epithelium and immune compartment, could function as one of the key cell types that regulate the organ homeostasis.

As one representative of mesenchymal stromal cell, colonic mesenchymal stromal cells reside underneath the colonic epithelium, covering lamina propria, submucosa, muscularis mucosae, muscularis propria, serosa and mesothelium. Those cells include telocytes (8), CD34^+^ fibroblasts (10), pericytes (11), Map3k2-Regulated Intestinal Stromal Cells (MRISCs) (12), myocytes (13), interstitial cell of cajal (14) and mesothelial cells (15). They are negative for hematopoietic lineage gene *PTPRC* (CD45), epithelial lineage gene *EPCAM* or *CDH1* (E-Cadherin), endothelial lineage gene *PECAM1*(CD31). Vimentin, CD90, S100A4, α-smooth muscle actin and desmin have been utilized to identify different subsets of mesenchymal stromal cell populations (1). Recently, with the introduction of single cell RNA sequencing and high-dimensional flow cytometry, multiple surface markers have been identified to characterize different subsets of colonic mesenchymal stromal cells including gp38^+^CD140a^lo^CD34^+^CD81^-^ fibroblasts (10), gp38^+^CD140a^lo^CD90^+^CD34^+^CD81^+^CD138^-^ MRISC (16), gp38^+^CD140a^Hi^CD34^-^CD201^+^ subepithelial telocytes (8), gp38^-^ CD146^+^ CD140b^+^ pericytes (17), gp38^+/-^CD146^+^ myocytes (17).

Currently, most studies on the biological function of intestinal mesenchymal stromal cell subsets have been focused on their physiological function. For example, interstitial cells of cajal, together with myocytes, regulate the motility and provide the mechanical support for the colon (18). Pericytes, on the other hand, surround the capillary vasculature to regulate vascular stretching and permeability. Fibroblasts labeled by CD34 and gp38 are considered to regulate intestinal homeostasis *via* producing Wnt ligands and agonists Wnts and R-spondins to support the intestinal stem cell niche (10). On the other hand, telocytes are considered to regulate epithelial cells *via* producing canonical and non-canonical Wnt ligands and BMPs (8). Recently, a novel sub-crypt stem cell niche supporter, namely trophocytes in small intestine (16) or MRISCs in colon (12) marked by CD81 have been identified as one of the key R-spondin and BMP antagonist provider for the intestinal stem cell niche. In one-word, mesenchymal stromal cell subsets help form a biochemical Wnt-BMP antagonizing gradient to facilitate the differentiation of intestinal epithelial cell and provide mechanical support for gut motility.

With the technological advancement of single cell RNA sequencing, a complete gene expression profile of intestinal mesenchymal stromal cell subsets has been revealed. These data indicated that intestinal mesenchymal stromal cells not only provide physiological support for gut epithelium, endothelium and musculature, but are also involved in organizing two sub-epithelial immune regulatory centers that can potentially interact with different types of immune cells in response to scenarios for either type II or type III immunity. In this mini-review, we will utilize those available single cell transcriptomic datasets and propose the model of two immune regulatory hubs alongside crypt-villus axis orchestrated by the heterogeneous mesenchymal stromal cell subsets.

## 2 Two immuno-regulatory hubs regulated by distinct mesenchymal stromal cell subsets

### 2.1 Heterogeneity of distinct mesenchymal stromal cell subsets

#### 2.1.1 CD34^+^CD81^-^ *Pdgfra* ^lo^ murine intestinal fibroblasts or stromal 1 in human

**Figure 1:**
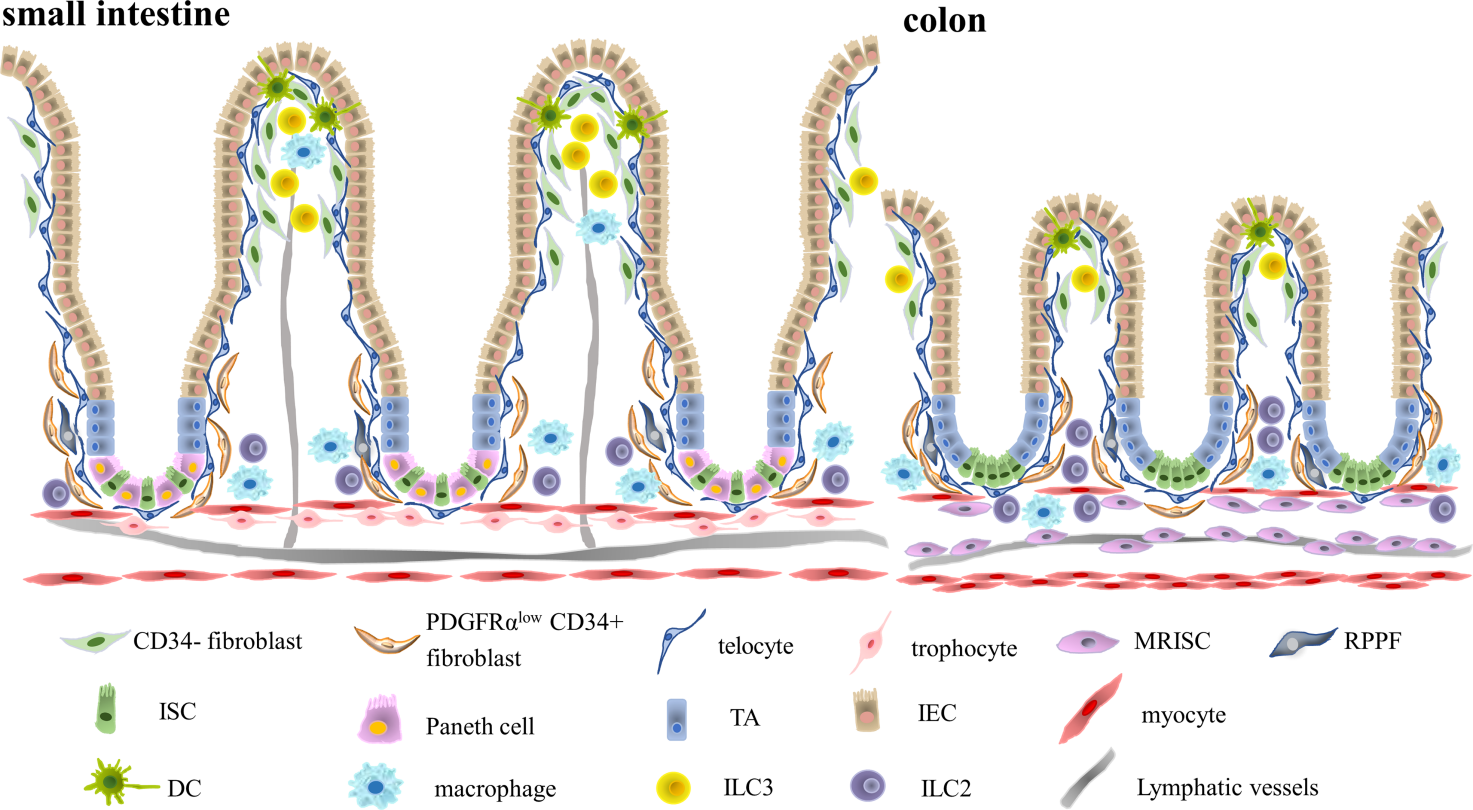
Two Distinctive Immune Niches Established by Multiple Different Intestinal Mesenchymal Stromal Cells. The intestinal stroma is composed of multiple distinctive intestinal mesenchymal stromal cell subsets including trophocytes, telocytes, fibroblasts and myocytes. Different subsets of intestinal mesenchymal stromal cells occupy different regions. Telocytes are located in the basal membrane right adjacent to the intestinal epithelium, while trophocytes are located in the submucosa. Intestinal fibroblasts are located in the lamina propria. Intestinal immune cells, on the other hand, also occupied different regions of intestinal stroma. CD11c^+^ dendritic cells mainly resided in the villus tip, while CD206^+^ macrophages occupied the lamina propria region. Type III innate lymphoid cells are situated in the villus tip and isolated lymphoid follicles, while type II innate lymphocytes are mainly enriched in the submucosa. Therefore, there might be potential interaction between villus tip intestinal stromal cells like fibroblasts or telocytes with CD11c^+^ dendritic cells in the crypt top and fibroblasts or trophocytes interaction with crypt bottom immune cells like CD206^+^ macrophages and type II innate lymphocytes. (MRISC, Map3k2-Regulated Intestinal Stromal Cell, RPPF, Rare Peri-cryptal Ptgs2-expressing Fibroblasts, TA, Transit Amplifying Cell, IEC, Intestinal Epithelial Cell, ISC, Intestinal Stem Cell, DC, Dendritic Cell, ILC3, Type III Innate Lymphoid Cell, ILC2, Type II Innate Lymphoid Cell).

The first identified group of intestinal mesenchymal stromal cell in mouse is named as CD34^+^ mesenchymal cells that located right near the intestinal crypt (10) (**Figure 1**). This unique group of intestinal mesenchymal stromal cell is in fact, also a heterogeneous population which contains at least two distinctive subsets. One group highly expresses CD90, which are localized near the intestinal crypt (19) (**Table 1**). Compared to CD90^negative^ mesenchymal stromal cells, these cells highly express intestinal stem cell niche factors like *Grem1*, *Rspo3* and *Wnt2b* (19). In addition, these CD90^+^ intestinal mesenchymal stromal cells specifically express Semaphorin 3 (19). *In vitro* co-culture of those CD90^+^ intestinal mesenchymal stromal cells with intestinal epithelial organoid promoted the spheroid formation while blockade of the semaphorin-neuropilin signal *via* exogenous recombinant neuropilin or knocking down neuropilin 2 could significantly interfere the spheroid formation (19). Therefore, this unique subset of intestinal mesenchymal stromal cell is one component which is responsible for the intestinal stem cell niche formation.

**Table 1:** Molecular phenotype and regulatory signatures of human and mouse intestinal mesenchymal stromal cell subsets.

Spatial Distribution	Mouse			Human			Ref		
	Cell Type Annotation	Marker (Lineage^-^)	TF	Cell Type Annotation	Marker (Lineage^-^)	TF				
Lamina Propria	Rare PGE2 Producing Fibroblast (RPPF)	gp38^+^CD146^-^CD140a^lo^CD34^+^ CD81^-^CD90^lo/-^FGFR2^+^	Runx2 Prdm1	Stromal 1	CD146^-^CD90^+^ ADAMDEC1^+^	RUNX2 FOXF1/2	(16, 17, 20)
Lamina Propria	CD34^+^Pdgfra^lo^ Fibroblast	gp38^+^CD146^-^CD140a^lo^CD34^+^CD81^-^CD90^Hi^	Isl1 Cebpa	Stromal 1	CD146^-^CD90^+^ CCL11^+^	CEBPA	(17, 20)
Sub-epithelium	Telocyte (Intestinal Subepithelial Myofibroblast)	CD140a^Hi^ CD201^+^ *Spon1^+^Foxl1^+^*	Foxl1	Stromal 2a	CD146^-^CD90^-^CD142^+^CPM^+^	FOXL1	(8, 17, 20)
		CD140a^Hi^ CD201^+^ *Foxl1^+^ Lgr5^+^Adamts18^+^*	Foxl1	Stromal 2b	CD146^-^CD90^-^CD142^+^BMP7^+^	FOXL1	(17, 19, 20)	
Underneath crypts, Submucosa	MRISC (Colon)	gp38^+^CD81^+^ CD90^+^CD34^+^ *Rspo1^+^*	Klf2 Ebf1	Stromal 3	CD146^-^CD90^+^CD34^+^ CD73^+^ RSPO3^+^GREM1^+^	FOXP2	(13, 17, 20)
	Trophocyte (SI)	gp38^+^CD81^+^ CD90^-^CD34^+^ *Grem1^+^*	Klf2 Ebf1				(11, 17, 20)	
Musculature	Myocyte	gp38^+^CD146^+^ *Cd59a^+^Pcp4^+^*	Mef2d Srf	Myocyte	CDH2^-^CD146^+^a9b1^+^ MFAP5^+^	MEF2 SRF	(17, 20)

Lineage, EpCAM/CD45/CD31; TF, Transcription Factors.

Murine FGFR2^+^ fibroblasts, on the other hand, express relatively low level of CD90 (21) (**Table 1**). One unique feature of those intestinal mesenchymal stromal cells is their capability in synthesizing prostaglandins. In *Apc* ^min^ mutation induced colorectal cancer model, intestinal mesenchymal stromal cell specific deficiency in PGE2 synthase would lead to fewer polyp formation (22). Such behavior is mediated by PGE2 specific stimulation of intestinal mesenchymal stromal cells *via* promotion of nuclear translocation of YAP protein (22). Therefore, intestinal fibroblasts could also be divided into two separate groups, Rare Peri-cryptal PGE2 producing fibroblasts (RPPF) and CD90^+^ crypt bottom fibroblasts. RPPFs are potentially regulated by RUNX2 or FOXF1 (23). Previous study has shown that Indian hedgehog signal could regulate the expression of *Foxf1* and *Foxf2* (24). Mice with defects in either *Foxf1* or *Foxf2* would lead to severe defect in colon villus elongation and downregulation of ECM component like Syndecan1 (24).

In human, on the other hand, those two groups of murine intestinal mesenchymal stromal cells are named as *CCL11* ^+^ and *ADAMDEC1* ^+^ stromal 1 cells (23, 25). *APOE*, *CCL8*, *FABP5* or *ADAMDEC1* are preferentially expressed by those cells. Extracellular matrix producing capability of those mesenchymal fibroblasts is also quite unique. They specifically produce collagens like COL14A1, COL15A1 or elastic fibers like fibronectin. SCENIC analysis also revealed that those cells are highly dependent on RUNX2 and PPARG (23).

#### 2.1.2 *Pdgfra* ^Hi^ *Foxl1* ^+^ murine telocytes or CD142^+^ human telocyte like cells

*Pdgfra* ^Hi^ *Foxl1* ^+^ telocytes are a group of spindle-shaped subepithelial myofibroblasts which are localized underneath the intestinal epithelium with long telopodes (8) (**Figure 1**). They express high level of Wnt ligands like Wnt2b, Wnt5a and Wnt inhibitors like soluble frizzled receptor proteins (i.e. *Sfrp1*), Dickkopf (i.e. *Dkk3*) and Wnt inhibitory factor (i.e. *Wif1*). On the other hand, they also express high level of BMP ligands like Bmp2, Bmp3, Bmp4, Bmp5, Bmp6, Bmp7. Depletion of intestinal telocytes *via* diphtheria toxin would lead to severe loss of intestinal villi (26). In addition, the expression of Foxl1^+^ telocyte specific Wnt ligand post-translational modifier Porcupine is responsible for the normal development of intestinal epithelial cells (27).

Recent spatial transcriptomic studies have shown that this seemingly homogeneous cell population in fact contains two distinctive subsets, one localized at the top of the villus, while the other localized in the bottom of the crypt (28). The villus tip telocytes express *Lgr5* and *Adamts18*. As compared to crypt bottom telocytes, villus tip telocytes highly express non-canonical ligands Wnt5a rather than canonical ligands Wnt2 or Wnt2b. Upon short-time depletion of villus tip telocytes using *Lgr5*-GFP-DTR system, the differentiation and maturation of intestinal epithelial cells have largely been affected, especially the villus tip feature of enterocytes like *Nt5e*, *Ada*, *Klf4* and *Cdh1* (28).

Another study has identified a villus tip telocyte expressing *Adamts18*, which promotes the integrity of villus tip vessel and epithelium (29). Depleting villus tip telocytes *via Lgr5*-DTA system would also affect the formation of villus tip endothelial cell network without affecting the maintenance of intestinal epithelial stem cell (29). Such function has been executed *via* VEGF-α (29). Crypt bottom telocytes, on the other hand, highly express F-spondin, Gremlin 1 and soluble Frizzled receptors (8). Therefore, telocytes could further be divided into at least two more subsets, namely *Lgr5* ^+^ villus tip telocytes and crypt bottom telocytes marked by *Spon1*.

In human, telocytes could also be identified using marker like CD142 (*F3*) (**Table 1**). Single cell transcriptomic studies have already annotated this unique type of cell as *SOX6* positive, specifically provides BMPs and non-canonical Wnt ligands like WNT5A and WNT5B (17). Human telocytes could also be separated into two groups, one specifically expressing *BMP7*, *WNT5A*, *LTBP1*, *PTX3* and *GJA1 (17*), while the other expressing *CPM (17*).

#### 2.1.3 CD81^+^ MRISC (trophocytes in small intestine) or type III human colonic stromal cell

One R-spondin1 expressing sub-population located near the colonic crypt marked by gp38, CD90, CD81, CD34 and with Rspo1 expression is called *Map3k2*-Regulated Intestinal Stromal Cell (MRISC) (12) (**Table 1** and **Figure 1**). Other studies on crypt bottom fibroblasts (30), trophocytes (16) or *Ackr4^+^* intestinal submucosal fibroblasts (31) have also shown similar functional features with MRISCs, though they were localized in different anatomical structures of the gastrointestinal tract. This unique type of stromal cells express different cytokines suggesting their potential immune-regulatory functions, including cytokines like M-CSF, IL-6 and IL-33 and chemokines like CCL-2, CCL-7 and CXCL-10 (12). They could also regulate intestinal stem cell niche *via* antagonizing BMP signal while promoting the Wnt signal by expressing *Grem1*, *Wnt2*, *Wnt2b*, *Wnt5a, Rspo1* and *Rspo3 (* 12). Upon co-culturing with intestinal organoid, they can promote the formation of more spheroids as compared to CD81^-^ intestinal mesenchymal stromal cells (12). Upon depletion of such cell type *via Grem1* initiated diphtheria toxin receptor, intestinal epithelial stem cells are largely diminished indicated by *Lgr5*, *Olfm4* and *Ki67* expression (16). Such biological function in promoting intestinal stem cell niche is to some extent, tightly regulated. Upon Dextran Sodium Sulfate induced mucosal damage, those cells would sense micro-environmental reactive oxygen species and increase the expression level of Wnt agonist R-spondin1 *via* ERK5 and KLF2 (12). In addition to the regulation of intestinal epithelial cells, this unique type of cell can also potentially regulate intestinal endothelium, by being strategically localized near the intestinal vessels and providing multiple different pro-angiogenesis factors like *Figf*, *Serpine1* and *Il6* (31). Therefore, those CD81^+^ intestinal mesenchymal stromal cells, by interacting with intestinal crypt niche components like endothelium and intestinal epithelial stem cells, lay the foundation of intestinal crypt niche.

In human, MRISC like cells could also be identified using marker like CD34, CD73 (*NT5E*) and CD90 (*THY1*) (23). Single cell transcriptomic studies have already annotated this unique type of cell as *FOXP2* positive, specifically provides BMP antagonists like GREM1 and Wnt agonists like RSPO3 (23).

Those three distinctive intestinal mesenchymal stromal cell populations altogether, help form the crypt-villus axis of intestinal subepithelial mesenchyme. In addition to those three subsets, the mouse colon also contains other mesenchymal stromal cell populations including myocytes and muscularis mucosae which are responsible for the formation of colon musculature.

#### 2.1.4 Gut musculature

The intestinal musculature is composed of multiple layers ranging from muscularis mucosae, which is mostly closed to the epithelium, myenteric plexus, longitudinal muscle, circular muscle to deep muscular plexus. The major cell type that is involved in the muscular regulation is myocyte. Those myocytes are *Actg2*, *Myh11* and *Des* positive, with the ability to provide the mechanical support for the intestine. Recent single cell transcriptomic studies have shown that intestinal myocytes are in fact also quite heterogeneous (13). There are at least three major types of intestinal myocyte like cells, which includes muscularis propria, lamina propria myocytes and muscularis mucosae. Among those cells, muscularis mucosae is localized underneath the intestinal crypt and provides the intestinal stem cell niche with BMP antagonists Gremlin 1, Gremlin 2 and Wnt agonists R-spondin 3. Other myocyte subsets like muscularis propria, lamina propria myocyte, on the other hand, also express BMP antagonizing intestinal epithelial stem cell niche factors like Noggin, Gremlin, Chordin like 1 and Wnt agonizing factors like R-spondin 3. Thus, intestinal myocytes, though locating underneath the intestinal crypt, also provide factors for intestinal epithelial stem cell maintenance and differentiation.

#### 2.1.5 Developmental origin of different intestinal mesenchymal stromal cell subsets

Before the invention of single cell transcriptomics, the developmental biologists used to rely on *in vivo* lineage tracing models or transplantation models to determine the developmental relationship in reality. Neural crest labeling system *Wnt1*-Cre, *Sox9*-Cre or *Mpz*-Cre could not trace to any adult intestinal mesenchymal stromal cell subpopulation (32), indicating that it is highly unlikely for adult intestinal mesenchyme to be derived from classical neural crest. Fate mapping using mesothelial cell specific *Wt1*-Cre has been traced to almost entire muscularis propria, indicating the major contribution of embryonic mesothelial cells to the gut musculature (15). Similar results could also be observed with another mesothelial cell specific tracking system using *Msln* as the driving promoter (32). Several other lineage tracing models have also been utilized to characterize lamina propria fibroblasts. *Foxl1* has been found to label a unique subset of intestinal subepithelial myofibroblasts, or telocytes (8), which could also be targeted by pericyte labeling system *Cspg4* (33). The downstream transcription factor of hedgehog signal, *Gli1* could be utilized to track most of the mesenchymal stromal cell populations except interstitial cell of cajal and mesothelial cells (34). *Grem1*-Cre could be utilized to track CD81^+^ trophocytes and other intestinal mesenchymal stromal cell populations within a year, indicating the potential precursor identity of CD81^+^ trophocytes (16). Recently, with the introduction of single cell transcriptomic studies, the heterogeneity and potential relationship among embryonic stromal cell subsets have been revealed. Results indicated that the heterogeneity of trophocyte like cells, telocyte like cells and myocytes may have already formed as early as the embryonic stage (35).

### 2.2 Distinctive immune niches established by heterogeneous stromal cell populations distributed in the colon

#### 2.2.1 Villus tip immune regulatory hub

The top of villus tip is the place where intestinal epithelial cells get contact with commensal bacteria. The top of villus tip is also the place where intestinal epithelial cells undergo anoikic type of cell death (36). To cope with such complicated environment filled with immunogens and quickly respond to the invading microbes, the capillary bed where leukocytes extravasated is also seated in the villus tip (29). Thus, the villus tip may form an immune hub enriched with multiple villus tip-resident cells including villus tip telocytes, dendritic cells and type III innate lymphocytes. The villus tip mesenchymal stroma could be the key organizer for this multi-cellular network due to their unique expression of adhesion molecules, chemokines and cytokines. The villus tip immune micro-environment is mainly organized by two different types of stromal cells, villus tip telocytes and gp38^+^CD34^-^ CD90^+^ fibroblasts.

The elongated villus tip telocytes marked by *Lgr5* are located at the top of the villus right underneath the intestinal epithelium, while gp38^+^CD34^-^ fibroblasts are located in the lamina propria surrounded by telocytes. Those two types of the mesenchymal stromal cells, together, can produce BMPs, IL-34 and CXCL14 to modulate and control the alternative activation and chemotaxis of macrophages. Previous studies have already shown that fibroblasts could secrete CXCL14 to stimulate monocyte migration and infiltration (37). In the tumor setting, CD163^+^ tumor-associated macrophage, on the other hand, could elicit cancer associated fibroblasts (CAFs) to express CXCL16, which could further expand these fibroblasts in an autocrine manner (38).

Stromal cells like gp38^+^CD34^-^ fibroblasts also play an important role in regulating ILC3 development and function (39). Those cells could provide metabolic supplements for the normal development of innate lymphocytes *via* oxysterol and prostaglandin production. Lymphoid tissue organizers in the fetus can differentiate into marginal reticular cells (MRC) and help form a stromal network for type III innate lymphocytes to reside in secondary lymphoid organs (40). Another study has also shown that intestinal mesenchymal stromal cells (MSCs) boosted the proliferation and IL-22 producing capabilities of ILC3s *via* IL-7 and aryl hydrocarbon receptor ligands expression (41). Such physiological function could be utilized to ameliorate graft-versus-host disease (GvHD) (41).

#### 2.2.2 Crypt bottom immune regulatory hub

The crypt bottom, however, is the place where active immune responses should be suppressed to ensure the protection of intestinal stem cell niche. Therefore, the major interacting partner in the crypt bottom is crypt fibroblast-macrophage pair and trophocyte-type II innate lymphocyte pair.

The interaction between fibroblast and macrophage plays an essential role in maintaining tissue homeostasis and disease progression. At steady state, fibroblast can secrete colony stimulating factor 1 (CSF1), which promotes survival and differentiation of macrophages *via* CSF1-CSF1R axis (42). On the other hand, Macrophages provide PDGFs for PDGFRα^+^ fibroblast, in such case PDGFRα can transduce *via* PI3K to activate an ATF3-mediated survival program in fibroblasts (43). In the setting of inflammation such as fibrosis, activated fibroblast produces CCL2 to attract monocyte or macrophage to lesion area (44). Activated macrophage can stimulate fibroblast to increase CSF1 production *in vitro* (44); increased PDGFs expression in macrophage promotes proliferation and fibrotic activation program in fibroblast (45). Transforming growth factor beta (TGFβ) and interleukin-6 (IL6) from macrophage or autocrine fibroblast drive fibroblast proliferation and activation (20, 46). In tumor micro-environment (TME), on the other hand, heterogeneous and functionally plastic cancer-associated fibroblasts (CAFs) are also involved in myeloid regulation. During the initiation and progression of cancer, the CAFs produce CCL2, recruit CCR2^+^ monocyte and macrophage to the tumors (47), CAFs and myeloid cell cooperatively promotes tumor growth. Complement C3 derived from CD34^+^ CAFs can recruit C3aR^+^ circulating monocytes to the TME (48).

Type II innate lymphoid cells (ILC2) are mostly seated near the colon adventitia and are involved in controlling the invasion of commensal and pathogenic microorganisms. Another important feature for those cells is wound repair and healing. Stromal cells within the local micro-environment are important for the function of ILCs. In Peyer’s patches and mesenteric lymph nodes, fibroblastic reticular cells (FRCs) were essential for maintaining type I innate lymphoid cells (ILC1) by producing of the cytokine IL-15 (49). Fetal liver derived ILC2 could also be supported by PDGFRα^+^ gp38^+^ mesenchymal cells for normal development (50). In addition, there are other studies on the regulation of ILC2 function by stromal cells in other tissues. In lung, adventitial stromal cells (ASCs), a fibroblast-like subset can support the accumulation and activation of ILC2 by releasing IL-33 and TSLP during helminth infection (51). In white adipose tissue, residential multipotent stromal cells can also function as a reservoir for IL-33. Upon cellular stress, those cells could help support the proliferative activity of LFA-1-expressing ILC2 (40). In pancreas, lipoprotein disorder induces interleukin-33 (IL-33) expression and release in pancreatic stellate cell (PSC), which strongly induced pancreatic ILC2s to trigger a type 2 immune response accompanied by the activation of PSCs, eventually leading to fibrosis during pancreatitis (52). Therefore, it has been expected that colonic mesenchymal stromal cells may possess similar functions in regulating colonic ILC2 function.

## 3 Mesenchymal stromal cells and intestinal diseases

Recent single cell transcriptomic studies have revealed the alteration of intestinal mesenchymal stromal cell diversity in multiple colonic diseases including inflammatory bowel diseases and colorectal cancer.

In ulcerative colitis, an inflammatory associated fibroblast was expanded with the expression of inflammation-related markers like IL-11, IL-24 and IL13RA2. This unique subset of intestinal mesenchymal stromal cell is actually quite heterogeneous, which expresses both crypt associated genes like WNT2B and villus associated genes like WNT5B. They also express series of cancer-associated fibroblast related genes like *FAP*, *TWIST1* and *WNT2*. A most critical feature of this inflammation associated fibroblast is the expression of *OSMR*, the receptor for oncostatin M, which predicts the anti-TNF therapy responses (53).

In Crohn’s diseases, on the other hand, the alteration of intestinal mesenchymal stromal cells are companied by the formation of ectopic lymphoid structures. Fetal mesenchymal lymphoid tissue organizer (mLTo) like T reticular cells and stromal 4 cells were expanded in Crohn’s disease patients (25). In colorectal cancer, there is also a dramatic change of the heterogeneity of intestinal mesenchyme. There is a significant down-regulation of BMP producing telocyte like cells and *CXCL14* ^+^ cancer associated fibroblasts in mismatch repair deficient (MMRd) patients as compared to mismatch repair proficient (MMRp) patients. The *RSPO3* ^+^ *GREM1* ^+^ intestinal mesenchymal stromal cells, however, were found in stromal bands that reached far upward from the base into the tumor body (54). The *FAP* ^+^ activated intestinal fibroblasts interact with *SPP1* ^+^ macrophages and form the desmoplastic barrier to ward off anti-tumor immune infiltration (23).

## 4 Discussion

In conclusion, recent single cell transcriptomic studies have revealed multiple distinctive fibroblast subsets that control intestinal epithelial cell differentiation *via* constructing Wnt and BMP antagonizing gradient. There are trophocyte like cells, fibroblasts, telocytes and myocytes in both human and mouse. Each of those four different intestinal mesenchymal stromal cell subtypes could further be separated into at least two distinctive subsets due to their localization in the gut tissues. Interdependency of distinctive intestinal mesenchymal stromal cells and immune cells help form crypt bottom and villus tip immune niches. Further studies are expected to demonstrate the immuno-regulatory function of each intestinal stromal cell subsets using novel *in vivo* lineage tracing models and *in vitro* culture of sorted cells based on newly identified markers deduced from single cell RNA sequencing results. Perturbation of such interaction could be utilized for next generation colitis and colorectal cancer treatment.

## Author contributions

HS, JT, HC, and NW wrote the manuscript and prepared the table and figure. BS planned, wrote and overall supervised the preparation of the manuscript. All authors read and approved the submitted version.

## Funding

This work was supported in part by grants from the National Natural Science Foundation of China (31930035, 91942311, 32061143028, 32170895, 82100575), Shanghai Science and Technology Commission (20410714000, 22ZR1480700, 22QA1408000) and National Key R&D Program of China (2021YFA1301400).

## Acknowledgments

We thank Yao Zhang for his initial help and suggestions in preparation of the manuscript. JT and HC are Zhiyuan honary PhD scholarship recipient. NW is recipient of Yu He Young Scholar Scholarship and HS is supported in part by Yu He Postdoctoral Scholarship. We also thank all the members of the Su laboratory for discussion and suggestions.

## Conflict of interest

The authors declare that the research was conducted in the absence of any commercial or financial relationships that could be construed as a potential conflict of interest.

## Publisher’s note

All claims expressed in this article are solely those of the authors and do not necessarily represent those of their affiliated organizations, or those of the publisher, the editors and the reviewers. Any product that may be evaluated in this article, or claim that may be made by its manufacturer, is not guaranteed or endorsed by the publisher.
